# Supplementary figures and images for: Leishmaniasis epidemiology in endemic areas of metropolitan France and its overseas territories from 1998 to 2020
Source: PLoS Negl Trop Dis. 2022 Oct 7;16(10):e0010745. doi: 10.1371/journal.pntd.0010745 (PMC9624409; doi:10.1371/journal.pntd.0010745)

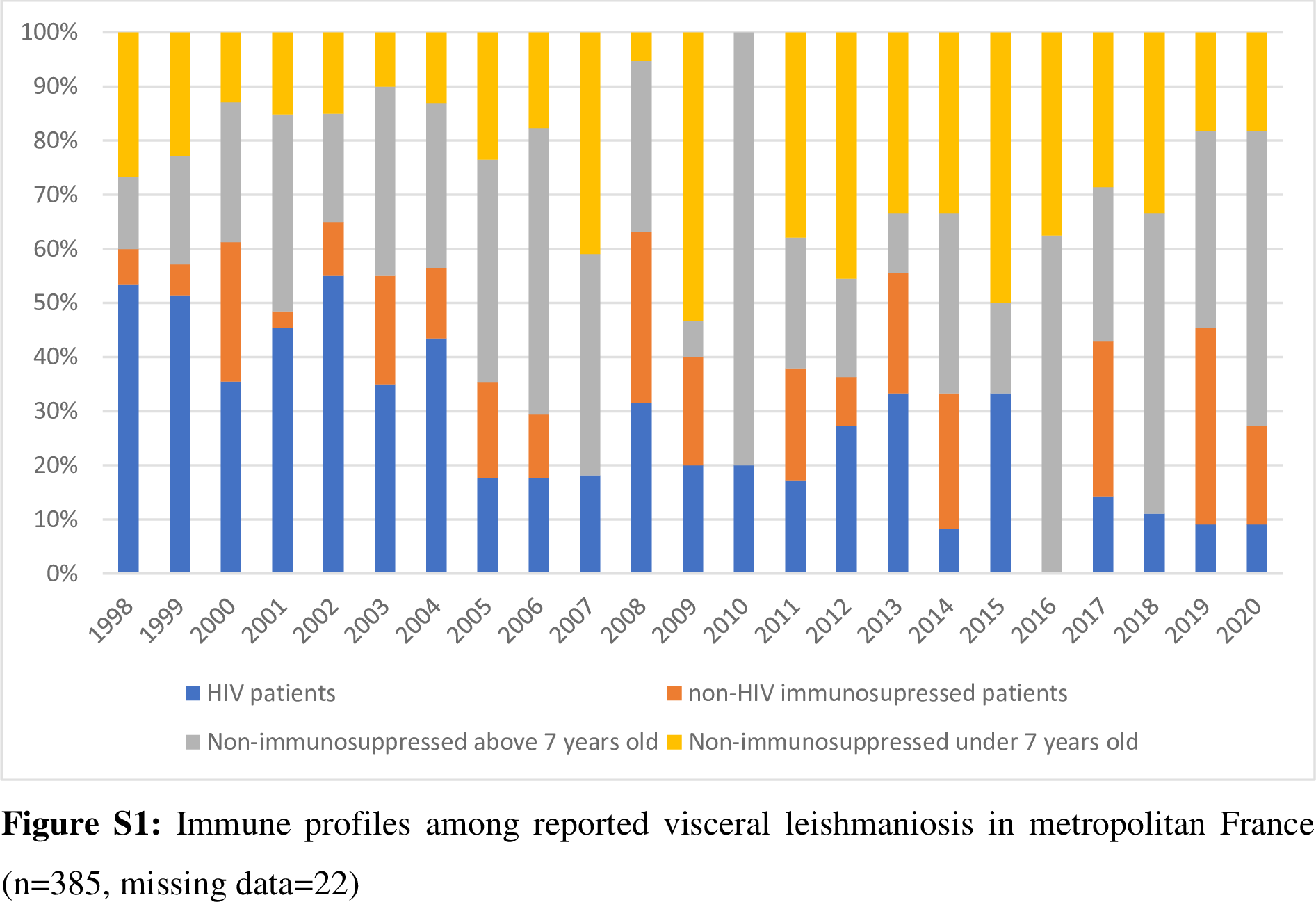

Supplement: S1 Fig — (TIF) [file pntd.0010745.s001.tif]

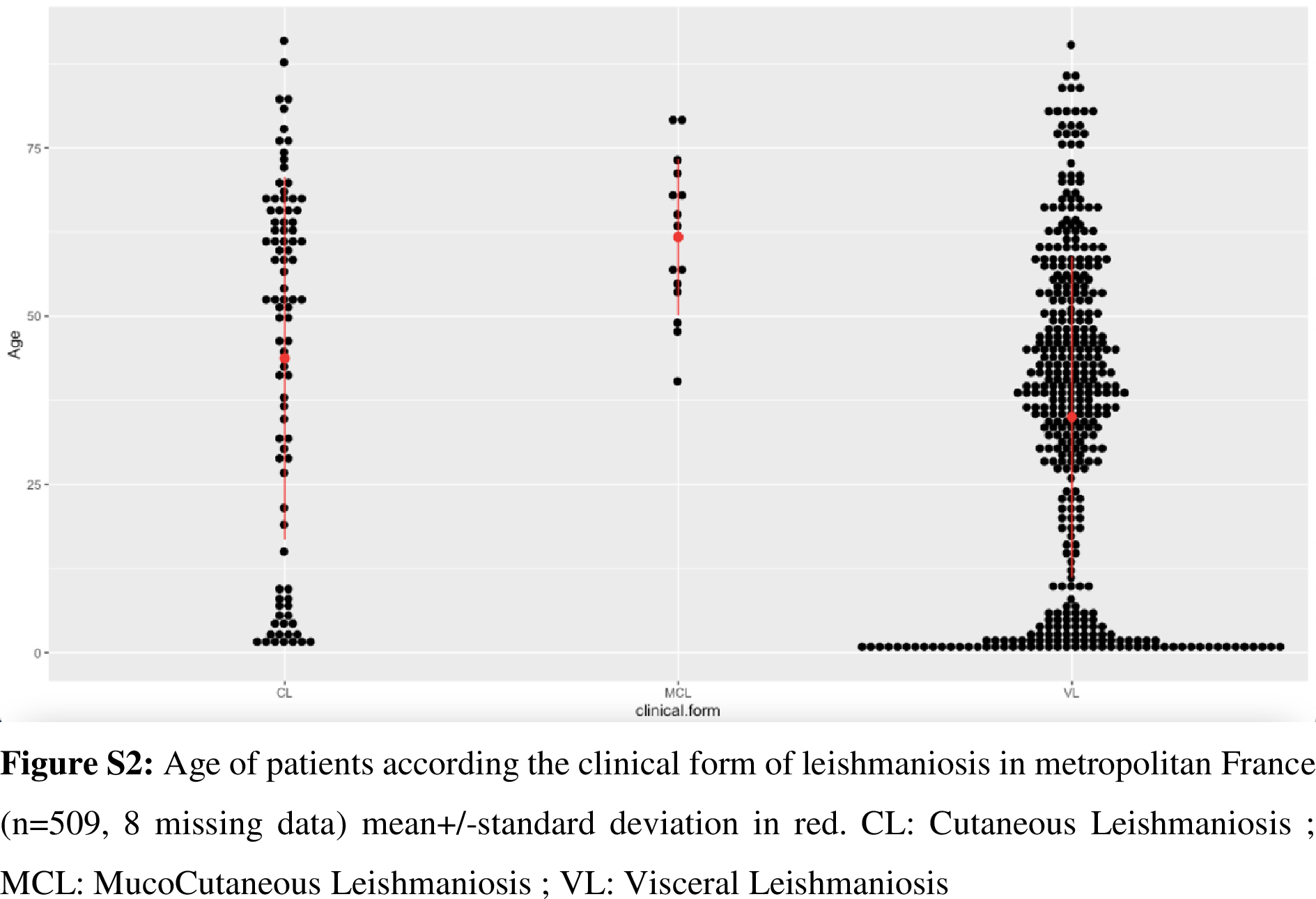

Supplement: S2 Fig — CL: Cutaneous Leishmaniasis; MCL: Muco-Cutaneous Leishmaniasis; VL: Visceral Leishmaniasis. (TIF) [file pntd.0010745.s002.tif]

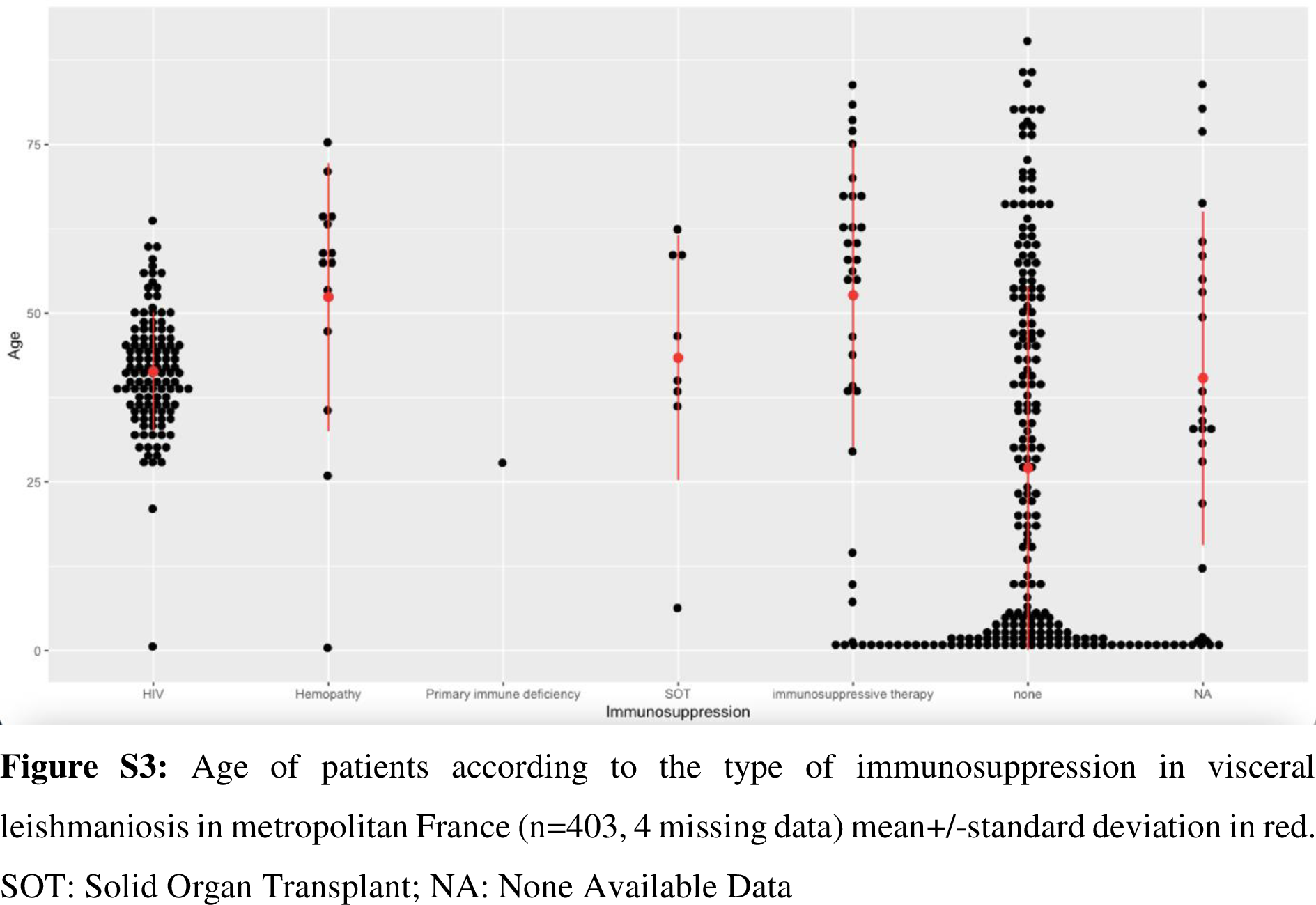

Supplement: S3 Fig — SOT: Solid Organ Transplant; NA: No data Available. (TIF) [file pntd.0010745.s003.tif]
