# Supplementary material for: Leishmaniasis epidemiology in endemic areas of metropolitan France and its overseas territories from 1998 to 2020
Source: PLoS Negl Trop Dis. 2022 Oct 7;16(10):e0010745. doi: 10.1371/journal.pntd.0010745 (PMC9624409; doi:10.1371/journal.pntd.0010745)
Supplement: S1 Table — (DOCX) [file pntd.0010745.s004.docx]

**S1 Table:** Number of unique and multiple lesions in cutaneous Leishmaniosis according to the species involved. N (%)

| **Old World** | | | |
| --- | --- | --- | --- |
| **species** | **unique lesions** | **multiple lesions** | **total** |
| *Leishmania infantum* | 104 (73) | 38 (27) | 142 |
| *Leishmania killicki* | 14 (74) | 5 (26) | 19 |
| *Leishmania major* | 191 (33) | 389 (67) | 580 |
| *Leishmania tropica* | 36 (59) | 25 (41) | 61 |
| **New World** | | | |
| **species** | **unique lesions** | **multiple lesions** | **total** |
| *Leishmania amazonensis* | 21 (68) | 10 (32) | 31 |
| *Leishmania braziliensis* | 97 (74) | 34 (26) | 131 |
| *Leishmania guyanensis* | 460 (50) | 453 (50 | 913 |
| *Leishmania lainsoni* | 8 (50) | 8 (50) | 16 |
| *Leishmania mexicana* | 9 (75) | 3 (25) | 12 |
| *Leishmania naiffi* | 11 (92) | 1 (8) | 12 |
