# Supplementary material for: Leishmaniasis epidemiology in endemic areas of metropolitan France and its overseas territories from 1998 to 2020
Source: PLoS Negl Trop Dis. 2022 Oct 7;16(10):e0010745. doi: 10.1371/journal.pntd.0010745 (PMC9624409; doi:10.1371/journal.pntd.0010745)
Supplement: S2 Table — (DOCX) [file pntd.0010745.s005.docx]

**S2 Table:** Number of lesions involving the face in cutaneous Leishmaniosis according to the species involved. N (%)

| **Old World** | | | |
| --- | --- | --- | --- |
| **species** | **Face lesion** | **No face lesion** | **total** |
| *Leishmania infantum* | 76 (59) | 52 (41) | 128 |
| *Leishmania killicki* | 10 (59) | 7 (41) | 17 |
| *Leishmania major* | 98 (20) | 396 (80) | 494 |
| *Leishmania tropica* | 30 (55) | 25 (45) | 55 |
| **New World** | | | |
| **species** | **Face lesion** | **No face lesion** | **total** |
| *Leishmania amazonensis* | 9 (32) | 19 (68) | 28 |
| *Leishmania braziliensis* | 24 (20) | 99 (80) | 123 |
| *Leishmania guyanensis* | 106 (13) | 729 (87) | 835 |
| *Leishmania lainsoni* | 1 (5) | 19 (95) | 20 |
| *Leishmania mexicana* | 6 (55) | 5 (45) | 11 |
| *Leishmania naiffi* | 0 (0) | 14 (100) | 14 |
