# Supplementary material for: Leishmaniasis epidemiology in endemic areas of metropolitan France and its overseas territories from 1998 to 2020
Source: PLoS Negl Trop Dis. 2022 Oct 7;16(10):e0010745. doi: 10.1371/journal.pntd.0010745 (PMC9624409; doi:10.1371/journal.pntd.0010745)
Supplement: Attachment — Submitted filename: Responses to the Reviewers.docx [file pntd.0010745.s006.docx]

**Reviewer's Responses to Questions**

Key Review Criteria Required for Acceptance?

As you describe the new analyses required for acceptance, please consider the following:

**Methods**

-Are the objectives of the study clearly articulated with a clear testable hypothesis stated?

-Is the study design appropriate to address the stated objectives?

-Is the population clearly described and appropriate for the hypothesis being tested?

-Is the sample size sufficient to ensure adequate power to address the hypothesis being tested?

-Were correct statistical analysis used to support conclusions?

-Are there concerns about ethical or regulatory requirements being met?

**Reviewer #1:** The study has a clear objective. However, the methods applied in data analysis do not support most of the discussions and conclusions carried out in the study. Authors must demonstrate their data in relation to the 95% Confidence Interval and not to one standard deviation. A Time Series Analysis must be performed so that statements related to possible trends observed in the study have statistical validity.

***Response***: *The statistical method was reviewed and modified with the help Pr Nicolas Nagot, professor of public health at the University Hospital of Montpellier. As you suggested, mean+/- standard deviation were changed into mean (95% Confidence interval). On your request, we performed Cochran-Armitage tests for trend to assess the changes in proportion and incidence over time. Eventually, we were able to demonstrate a significant decrease in incidence of leishmaniasis in metropolitan France (p<0.001) and in French Guiana (p<0.001) as well as a significant increase in imported cases (p<0.001). (cf Material & methods)*

**Reviewer #2:** -Are the objectives of the study clearly articulated with a clear testable hypothesis stated?

Yes

-Is the study design appropriate to address the stated objectives?

Yes

-Is the population clearly described and appropriate for the hypothesis being tested?

Yes

-Is the sample size sufficient to ensure adequate power to address the hypothesis being tested?

Yes

-Were correct statistical analysis used to support conclusions?

Yes

-Are there concerns about ethical or regulatory requirements being met?

Yes

**Reviewer #3:** This article aims to update the epidemiological situation of leishmaniasis in metropolitan France and its overseas territories. The authors used existing passive surveillance system established for indigenous and imported leishmaniasis cases in the country. The statistical methods used to analyze the available data and address the objectives of the study is appropriate.

--------------------

**Results**

-Does the analysis presented match the analysis plan?

-Are the results clearly and completely presented?

-Are the figures (Tables, Images) of sufficient quality for clarity?

**Reviewer #1:** The results are well presented, but they are mostly described data, without entering into some kind of spatio-temporal pattern. Thus, many discussions are not supported by the data presented.

Ex1: This may be due to new migratory dynamics and/or to the improvement of the notification system. Sojourns in the Maghreb region were at the origin of 53% (n=910) of imported cases (53%, n=910).

Ex2: A multicentric prospective study (28) reported that Brazilians represented 59.3% of infected people, mostly gold miners. This could explain the men to women sex ratio of 4.15 found in French Guiana compared with 1.89 in metropolitan France.

***Response***: *Indeed, examples 1 and 2 that you gave are not supported by the data presented because we did not exhaustively collect migration status; there is also the bias of underreporting of the reporting system (for example 1) and of not systematically reporting the occupation (for example 2). Nevertheless, these two sentences were only hypotheses to find leads that should be explored in future studies. These two sentences have been reworded to emphasize their hypothetical nature (lines 316 and 337).*

The study states that a reduction in incidence from 0.48 to 0.1 is a slight reduction. Depending on the graphic scale, it may seem small, but in proportional terms it is a reduction of 4.8 times. Is it statistically different? The time series decomposition could state whether the trend is decreasing or stability.

***Response***: *Concerning the decreasing incidence of leishmaniasis in metropolitan France, as you pointed out, we assessed statistically its evolution over time thanks to a Cochran-Armitage tests for trend. It was significant for the decreasing alternative hypothesis (p<0.001) as well as for the two-sided hypothesis (p<0.001) and not significant for the increasing alternative hypothesis (p=1).* Line 196.

**Reviewer #2:** - Does the analysis presented match the analysis plan?

Yes

-Are the results clearly and completely presented?

The reviewer suggests an implementation on the presentation of results,

***Response***: *For a good understanding, missing data were specified in the manuscript.*

-Are the figures (Tables, Images) of sufficient quality for clarity?

Yes

**Reviewer #3:** The results and analyses are clearly presented.

--------------------

**Conclusions**

-Are the conclusions supported by the data presented?

-Are the limitations of analysis clearly described?

-Do the authors discuss how these data can be helpful to advance our understanding of the topic under study?

-Is public health relevance addressed?

Reviewer #1: Many of the conclusions and discussions of the study are made from data that were not generated by the study or are not supported by the data presented. For example: This is related to the return from holidays in this region.

***Response****: You are right, the paragraph was rewritten in a hypothetical way as following (line 350 to 354): « The first hypothesis is that we have improved the reporting system, the second is that it is possible that there have been changes in migration flows, but we do not have data on this subject. Stays in the Maghreb accounted for 53% (n=910) of imported cases, another hypothesis would be the increase in vacation trips to this region. »*

The authors make the following statement: Moreover, cases showed a spatial heterogeneity.

What result or analysis can support this discussion? Only the visualization of maps would not allow this statement. Clustering analyses, such as Moran's Index, Moran's Local Index or spatial scanning should be used, if any discussion related to heterogeneity wants to be carried out.

***Response***: *As you suggest we performed Moran’s Index to assess the spatial heterogeneity of the leishmaniasis incidence in concerned french departements and in municipalities of French Guiana. No spatial autocorrelation was demonstrated among french departements (p=0.4) and municipalities of French Guiana (p=0.23). Thus, the following sentence was deleted («Moreover, cases showed a spatial heterogeneity»). Nethertheless, in French Guiana, most of the cases came from less populated inland municipalities (1736 cases for 92 506 inhabitants) rather than from more populated coastal areas (457 cases for 183 622 inhabitants) (Chi square test p<0.001). As a consequence, we modified the next sentence as followed: « Moreover, most of cases were reported from inland localities, although the population lives mainly along the coastal area (p<0.001). » (lines 366-368)*

Second paragraph of the discussion, at the beginning only reaffirms results, without discussing.

***Response***: *The three first sentences of the second paragraph were rephrased to emphasize the discussion about the decreasing incidence of leishmaniasis in the metropolitan France.*

Page 19: Concerning the clinical features, L. guyanensis was more often associated with multiple cutaneous lesions than L. braziliensis, as mentioned elsewhere (28). Review the sentence. In some parts of the text the percentage value - such as 10%, is displayed twice.

***Response****: As demanded the following sentence was reviewed: « As previously reported (28), patients infected by L. guyanensis were more likely to develop multiple lesions compared to those infected by L. braziliensis. ». Line 323, the supernumerary 10% has been removed.*

The authors make the following statement: Nevertheless, the analysis of data over a long period of time allowed a reliable assessment of the disease spatiotemporal changes. A descriptive analysis of the data does not support this result. The temporal analysis was discussed in a descriptive way. And the spatial analysis was performed only with thematic maps. A spatiotemporal pattern analysis should be conducted.

***Response****: As you suggested, temporal analysis of incidence was assessed by Cochrane Amittage test for trend and spatial analysis of incidence was performed thanks to Moran’s index to determine spatial clustering. Despite this, we agree with you to acknowledge that we mainly performed a descriptive analysis and thus we reworded this conclusion sentence (line 365): « Nevertheless, the analysis of national data over a long period of time has updated and shed new light on the epidemiology of leishmaniasis in France.”*

**Reviewer #2:** -Are the conclusions supported by the data presented?

Yes

-Are the limitations of analysis clearly described?

Yes

-Do the authors discuss how these data can be helpful to advance our understanding of the topic under study?

Yes

-Is public health relevance addressed?

Yes

**Reviewer #3:** The conclusion is supported by the data and analysis. The limitation of the data and analysis is clearly described. The paper also pinpoints the potential use of the presented information for public health measures.

--------------------

**Editorial and Data Presentation Modifications?**

Use this section for editorial suggestions as well as relatively minor modifications of existing data that would enhance clarity. If the only modifications needed are minor and/or editorial, you may wish to recommend “Minor Revision” or “Accept”.

**Reviewer #1:** There is a need for changes in methodology and analysis, so that the results are more clearer and the study discussions are related to its findings. Therefore, my opinion is for the rejection of the paper, requesting that changes be made in the structure of the document, for a future new submission in the journal.

***Response****: We have adapted our methodology, taken into account your constructive suggestions and moderated our conclusions which were not supported by the presented data and were therefore only assumptions.*

**Reviewer #2:** (No Response)

**Reviewer #3:** This paper presents important epidemiological updates about the status of leishmaniasis (Cutaneous; Mucocutaneous and visceral forms), parasite species, demographic characteristics and their distributions despite the limitedness of data availability and use of a passive surveillance data. The authors may consider the following minor edits:

The term Tegumentary leishmaniais is less commonly used and not very well understood especially in the old world. Therefore, please consider replacing by cutaneous and mucocutaneous leishmaniasis where ever it's used in the manuscript.

***Response****: The four occurrences of « tegumentary leishmaniasis » has been changed to « cutaneous and mucocutaneous leishmaniasis » as you recommended.*

The leishmaniasis situation in traditionally non-endemic areas of metropolitan France has not been highlighted or discussed; however, as the paper attempts to discuss expansion of leishmaniasis, its important to include some updates in this regard, e.g., reports of leishmaniasis in the Jura region…

***Response****: Thank you for this interesting suggestion. A sentence has been added in the discussion possible expansion of the endemic zone in France (line 334_335).” Some authors have warned of possible transmission of leishmaniasis outside Mediterranean coast such as in the Jura (27) but it remained exceptional.”*

Place of contamination ...in this paper is used as place of origin of infection or disease. Whereas in English as this could be confusing and as its commonly used, it's preferred to use ..Place where infection or disease acquired, Place where infection/disease contracted or place of transmission;

***Response****: « Place of contamination » has been changed to « Place of transmission » as you recommended.*

Please replace Near East by middle east or the exact countries referred as this term is not standard and no more used and may not be understood by many readers of the article/journal.

***Response****: « Near East » was rephrased as « Eastern Mediterranean countries » and the involved countries were specified after the first occurrence in the text.*

For the titles of the figures, e.g., Fig 4, 5 .. consider replacing 'repartition' by 'distribution' ....

***Response****: « Repartition » has been replaced by « distribution » in the manuscript.*

On page 18, last sentences of the second paragraph, please consider rephrasing as follows : These data could be used to revise treatment protocols and .....

***Response****: The sentence has been rephrased as you suggested.*

--------------------

**Summary and General Comments**

Use this section to provide overall comments, discuss strengths/weaknesses of the study, novelty, significance, general execution and scholarship. You may also include additional comments for the author, including concerns about dual publication, research ethics, or publication ethics. If requesting major revision, please articulate the new experiments that are needed.

**Reviewer #1:** The greatest strength of the study is a large database, with very important information in the understanding of leishmaniasis. The weakness of the study is that it does not apply a methodology that supports the discussions presented.

***Response****: As you recommended, we have adapted the methodology and changed conclusions as mentioned above.*

**Reviewer #2:** The manuscript on the "Leishmaniasis epidemiology in endemic area of metropolitan France and its overseas territories from 1998 to 2020" is very interesting and well structured. However, the presentation of data and its analysis sometimes appears incorrect because the missing /incomplete data are not reported. For the section Results missing data are reported only in the Supporting Information. For this the reviewer proposes to improve methods and results.

The manuscript is for “Minor Revision”.

***Response****: As you recommended, missing data were specified in the manuscript.*

Specific comments

Pag. 4, Line15: Correct “The animal reservoir host…” in “The principal animal reservoir host…”.

***Response****: Thank you for this comment. The text was corrected.*

Pag. 6, Lines 8-16: The AA should briefly summarise the methods used for human diagnosis and Leishmania species identification. The link for the reporting form is important but it is difficult to the readers look for the specific diagnostic details and methods applied.

***Response****: the following paragraph was added in material and methods: “Biological diagnosis was performed according to the protocols in use in each center: direct examination, culture, PCR (and serology for VL). Species identification was based on MLEE or molecular method or MALDI-TOF.”*

Pag. 6, Lines 16-19: As suggested by WHO, diagnosis of VL and CL are different. VL diagnosis is a combination of a parasitological (microscopic, culture or molecular) and a serological technique. In addition, the AA don’t give any information about the diagnostic gene/s primers for real-time PCR.

***Response****: Various technics for Leishmania detection were used according to the reporting laboratories and the clinical form of Leishmaniasis. It is therefore difficult to summarize all the details in the "methods" section and out of our focus. For Leishmania species identification, various technics were also applied according the time and the place of diagnostic : isoenzymatic methods^1^, mass-spectrometry (MALDI-TOF)^2^ and molecular analyses^3^.*

1) *PRATLONG, F., LAMI, P., RAVEL, C., BALARD, Y., DEREURE, J., SERRES, G., . . . DEDET, J. (2013). Geographical distribution and epidemiological features of Old World Leishmania infantum and Leishmania donovani foci, based on the isoenzyme analysis of 2277 strains. Parasitology, 140(4), 423-434. doi:10.1017/S0031182012001825*
2) *Lachaud L, Fernández-Arévalo A, Normand AC, et al. Identification of Leishmania by Matrix-Assisted Laser Desorption Ionization-Time of Flight (MALDI-TOF) Mass Spectrometry Using a Free Web-Based Application and a Dedicated Mass-Spectral Library. J Clin Microbiol. 2017;55(10):2924-2933. doi:10.1128/JCM.00845-17*
3) *Van der Auwera G, Bart A, Chicharro C, et al. Comparison of Leishmania typing results obtained from 16 European clinical laboratories in 2014. Euro Surveill. 2016;21(49):30418. doi:10.2807/1560-7917.ES.2016.21.49.30418*

Results: In the whole section missing data are not reported in the manuscript text except in the Supporting Information. The AA should improve the manuscript text furnishing both missing and incomplete data in the results. Similarly, the total of Leishmania species identification samples (strains and bioptic samples) should be indicated.

***Response****: Missing data were detailed in figures titles when it was needed. In the main text, each proportion was also expressed as an absolute number so that missing data could be inferred from a careful reading. This allowed not to overload the main text.*

Pag. 9, Lines 4 and 13: the geographical term of Near East is often overlapping with Middle East. Middle East is the geographical area better identified therefore the AA should clarify (as reported in the map) regions remaining in the Near East.

***Response****: The « Near East » term has been replaced by « Eastern Mediterranean countries » and the countries concerned have been made explicit in the text.*

Refs 16 and 17. The AA should check these 2 references because they appear reverse as citation in the text (Pages 9 and 10).

***Response***: Thank you for your vigilance. Indeed, the citations were inverted. This has been corrected in the text and in the bibliography.

**Reviewer #3:** This article aims to update the epidemiological situation of leishmaniasis in metropolitan France and its overseas territories. Considering the limitation of the passive surveillance, the paper has analysed and presented the trend in the three forms of the disease over the years, the distribution of cases and parasite species over the geographic areas focusing mainly on the endemic areas and available demographic variables. As the paper attempts also to discuss the potential expansion of the disease with changing environmental and climatic changes, its important to include some highlights/reports from non-endemic areas and also to elaborate on how or what type of active surveillance can be implemented as this is one of their final conclusion and recommendation.

***Response****: In the discussion, a sentence was added regarding leishmaniasis cases from non-endemic areas, and another sentence was added to clarify the type of active surveillance that could be implemented.*

--------------------

Figure Files:

While revising your submission, please upload your figure files to the Preflight Analysis and Conversion Engine (PACE) digital diagnostic tool, [https://urldefense.com/v3/__https://pacev2.apexcovantage.com__;!!DV4KuIgKKrh48VMFxQ!Xz7JzVs5IRxb0XRl8zVMLKm40cbLzUGDJhb6TewK_6DLxfXrZ-GDfLR6-1YXM9jiXf-GSXfVlDjG$](https://urldefense.com/v3/__https:/pacev2.apexcovantage.com__;!!DV4KuIgKKrh48VMFxQ!Xz7JzVs5IRxb0XRl8zVMLKm40cbLzUGDJhb6TewK_6DLxfXrZ-GDfLR6-1YXM9jiXf-GSXfVlDjG$) . PACE helps ensure that figures meet PLOS requirements. To use PACE, you must first register as a user. Then, login and navigate to the UPLOAD tab, where you will find detailed instructions on how to use the tool. If you encounter any issues or have any questions when using PACE, please email us at [figures@plos.org](mailto:figures@plos.org).

Data Requirements:

Please note that, as a condition of publication, PLOS' data policy requires that you make available all data used to draw the conclusions outlined in your manuscript. Data must be deposited in an appropriate repository, included within the body of the manuscript, or uploaded as supporting information. This includes all numerical values that were used to generate graphs, histograms etc.. For an example see here: [https://urldefense.com/v3/__http://www.plosbiology.org/article/info*3Adoi*2F10.1371*2Fjournal.pbio.1001908*s5__;JSUlIw!!DV4KuIgKKrh48VMFxQ!Xz7JzVs5IRxb0XRl8zVMLKm40cbLzUGDJhb6TewK_6DLxfXrZ-GDfLR6-1YXM9jiXf-GSdCIseKS$](https://urldefense.com/v3/__http:/www.plosbiology.org/article/info*3Adoi*2F10.1371*2Fjournal.pbio.1001908*s5__;JSUlIw!!DV4KuIgKKrh48VMFxQ!Xz7JzVs5IRxb0XRl8zVMLKm40cbLzUGDJhb6TewK_6DLxfXrZ-GDfLR6-1YXM9jiXf-GSdCIseKS$) .

Reproducibility:

To enhance the reproducibility of your results, we recommend that you deposit your laboratory protocols in [https://urldefense.com/v3/__http://protocols.io__;!!DV4KuIgKKrh48VMFxQ!Xz7JzVs5IRxb0XRl8zVMLKm40cbLzUGDJhb6TewK_6DLxfXrZ-GDfLR6-1YXM9jiXf-GSSRdprRu$](https://urldefense.com/v3/__http:/protocols.io__;!!DV4KuIgKKrh48VMFxQ!Xz7JzVs5IRxb0XRl8zVMLKm40cbLzUGDJhb6TewK_6DLxfXrZ-GDfLR6-1YXM9jiXf-GSSRdprRu$) , where a protocol can be assigned its own identifier (DOI) such that it can be cited independently in the future. Additionally, PLOS ONE offers an option to publish peer-reviewed clinical study protocols. Read more information on sharing protocols at [https://urldefense.com/v3/__https://plos.org/protocols?utm_medium=editorial-email&utm_source=authorletters&utm_campaign=protocols__;!!DV4KuIgKKrh48VMFxQ!Xz7JzVs5IRxb0XRl8zVMLKm40cbLzUGDJhb6TewK_6DLxfXrZ-GDfLR6-1YXM9jiXf-GSVlihxeq$](https://urldefense.com/v3/__https:/plos.org/protocols?utm_medium=editorial-email&utm_source=authorletters&utm_campaign=protocols__;!!DV4KuIgKKrh48VMFxQ!Xz7JzVs5IRxb0XRl8zVMLKm40cbLzUGDJhb6TewK_6DLxfXrZ-GDfLR6-1YXM9jiXf-GSVlihxeq$)

__________________________________________________

In compliance with data protection regulations, you may request that we remove your personal registration details at any time. (Use the following URL: [https://urldefense.com/v3/__https://www.editorialmanager.com/pntd/login.asp?a=r__;!!DV4KuIgKKrh48VMFxQ!Xz7JzVs5IRxb0XRl8zVMLKm40cbLzUGDJhb6TewK_6DLxfXrZ-GDfLR6-1YXM9jiXf-GSSTfLQD4$](https://urldefense.com/v3/__https:/www.editorialmanager.com/pntd/login.asp?a=r__;!!DV4KuIgKKrh48VMFxQ!Xz7JzVs5IRxb0XRl8zVMLKm40cbLzUGDJhb6TewK_6DLxfXrZ-GDfLR6-1YXM9jiXf-GSSTfLQD4$) ). Please contact the publication office if you have any questions.
